# *Mauritia flexuosa* Presents *In Vitro* and *In Vivo* Antiplatelet and Antithrombotic Activities

**DOI:** 10.1155/2013/653257

**Published:** 2013-12-18

**Authors:** Eduardo Fuentes, Wilson Rodríguez-Pérez, Luis Guzmán, Marcelo Alarcón, Simón Navarrete, Oscar Forero-Doria, Iván Palomo

**Affiliations:** ^1^Department of Clinical Biochemistry and Immunohematology, Faculty of Health Sciences, Interdisciplinary Excellence Research Program on Healthy Aging (PIEI-ES), Universidad de Talca, Talca, Chile; ^2^Centro de Estudios en Alimentos Procesados (CEAP), CONICYT-Regional, Gore Maule, R09I2001 Talca, Chile; ^3^Facultad de Ciencias Básicas, Universidad de la Amazonia, Florencia, Colombia; ^4^Chemical Institute of Natural Resources, Universidad de Talca, Talca, Chile

## Abstract

Fruit from the palm *Mauritia flexuosa* is one of the most important species in Peru, Venezuela, Brazil, Colombia, Bolivia, and Guyana. The present study aimed to investigate the antiplatelet and antithrombotic activities of oil extracted from *Mauritia flexuosa*. The fatty acid contents were determined by gas chromatography—mass spectrometry. Oil extract of peel of *Mauritia flexuosa* was extracted by soxhlet extraction. The oil extract inhibited platelet secretion and aggregation induced by ADP, collagen, and TRAP-6 by a concentration-dependent way (0.1 to 1 mg/mL) without the participation of the adenylyl cyclase pathway and diminished platelet rolling and firm adhesion under flow conditions. Furthermore, the oil extract induced a marked increase in the rolling speed of leukocytes retained on the platelet surface, reflecting a reduction of rolling and less adhesion. At the concentrations used, the oil extract significantly decreased platelet release of sP-selectin, an atherosclerotic-related inflammatory mediator. Oil extract inhibited thrombus growth at the same concentration as that of aspirin, a classical reference drug. Finally, the data presented herein also demonstrate for the first time to our knowledge the protective effect of oil extracted from *Mauritia flexuosa* on platelet activation and thrombosis formation.

## 1. Introduction

Platelets play an essential role in the pathogenesis of cardiovascular diseases (CVD) [1]. In this way, the antiplatelet therapy has been used for a long time in an effort to prevent CVD [2].

Diets rich in fruits and vegetables (F&V), part of the so-called Mediterranean diet, reduce blood pressure and the risk of adverse cardiovascular events [3]. In addition, the regular consumption of fruits and vegetables has been shown to be beneficial in terms of reducing the platelet activity [4, 5]. Thus, in primary prevention trials, an energy-unrestricted Mediterranean diet supplemented with extra-virgin olive oil or nuts resulted in a substantial reduction in the risk of major cardiovascular events among high-risk persons [6]. Even, a higher consumption of vegetable oils rich in monounsaturated and polyunsaturated fatty acids (sunflower, corn, canola, and olive oils) has been associated with lower concentrations of inflammatory mediators [7], oxidative damage [8], and an increase of the healthy cholesterol (HDL cholesterol) [9].

Of the estimated 30 million species of plants found in the Amazon, only a few have been studied and identified so far, based on popular knowledge and scientific studies. As the use of Amazonian vegetable oils has increased in recent years, it is important to acquire a better knowledge of their properties in order to optimize the use of these materials [10]. The burití, moriche, canangucha, mirití, or aguaje (*Mauritia flexuosa*) is a species of palm belonging to the Arecaceae family. It is one of the most important of tropical America, found in Peru, Venezuela, Brazil, Colombia, Bolivia, and Guyana. Fruit from the palm *Mauritia flexuosa* is harvested for subsistence and commercial purposes [11]. Oil extracted from fruits of *Mauritia flexuosa is *used in cooking and is rich in monounsaturated fatty acids [12] and natural antioxidants [13]. Also, *Mauritia flexuosa* oil has found applications in the cosmetic industry due to its emollient properties and can be used as an adjuvant in sun protection [14]. Yet, the protective effect of *Mauritia flexuosa* on platelet activation and thrombosis formation remains to be fully elucidated. The present study aimed to investigate the antiplatelet and antithrombotic activities of oil extract from peel of fruits of *Mauritia flexuosa*.

## 2. Materials and Methods

### 2.1. Reagents

Sodium chloride (p.a.) was obtained from Arquimed (Santiago, Chile). Adenosine 5′-diphosphate (ADP), thrombin receptor activator peptide 6 (TRAP-6), calcein-AM, collagen, acetylsalicylic acid (ASA), bovine serum albumin (BSA), SQ22536 (an adenylyl cyclase inhibitor), rose bengal, prostaglandin E_1_ (PGE_1_), rhodamine 6G, and dimethyl sulfoxide (DMSO) were obtained from Sigma-Aldrich (St. Louis, MO, USA). Luciferase-luciferin reagent was obtained from Chrono-Log corp (Havertown, PA) and microfluidic chambers were from Bioflux (Fluxion, San Francisco, CA, USA).

### 2.2. Vegetable Oil

For the Soxhlet extraction we used a peel from fruits of *Mauritia flexuosa* purchased from local markets in Colombia. The peel was separated from the pulp in a kitchen food processor, washed with water, and frozen until used in order to avoid any modification due to exposure to heat or light. Then, the peel was subjected to Soxhlet extraction using hexane as a solvent for 8 hours, removing the solvent by distillation under reduced pressure at 37°C. After the evaporation of the solvent, yellow, limpid oil was obtained (yield 13%). The oil extract from *Mauritia flexuosa* was frozen until it was used. For *in vitro* and *in vivo* studies the oil sample was mixed homogenously in DMSO 0.3%.

### 2.3. Fatty Acid Determination by Gas Chromatography

The fatty acid profile was determined as fatty acid methyl esters by gas chromatography-mass spectrometry. The methyl esters were prepared using the method described by Morrison and Smith [15]. Separation of fatty acid esters was performed using an Agilent 7890A Gas Chromatograph equipped with an Agilent J&W DB-5ms capillary column (30 m × 0.32 mm × 0.5 *μ*m). The column temperature was programmed at 100°C for 1 min, then it was increased to 200°C at 5°C/min, 250°C at 3°C/min, and 300°C at 5°C/min. Helium was used as carrier gas with constant linear velocity of 14 mL/min. The injector temperature was set at 150°C, in splitless mode. The temperature of the mass spectrometer detector quadrupole was 150°C and for the source 230°C, in an acquisition mode of scan.

### 2.4. Preparation of Human Platelet Suspensions

Venous blood samples were taken from two young healthy volunteers—who previously signed informed consents—in 3.2% citrate tubes (9 : 1 v/v) by phlebotomy with vacuum tube system (Becton Dickinson Vacutainer Systems, Franklin Lakes, NJ, USA). The protocol was authorized by the ethic committee of Universidad de Talca in accordance with the Declaration of Helsinki (approved by the 18th World Medical Assembly in Helsinki, Finland, 1964). Tubes were centrifuged (DCS-16 Centrifugal Presvac RV) at 240 g for 10 minutes to obtain platelet-rich plasma (PRP). PRP was adjusted to 200 × 10^9^ platelets/L with platelet-poor plasma (PPP) obtained by centrifugation of the original tubes at 650 g (10 minutes). Washed platelets were prepared in Tyrode-HEPES buffer containing 50 ng/mL PGE_1_ and 1 mmol/L ACD-A, pH 7.4, at a concentration of 200 × 10^9^ platelets/L. Platelet counts were performed in a hematologic counter (Bayer Advia 60 Hematology System, Tarrytown, NY, USA).

### 2.5. Assessment of Platelet Activation by Quantization of Soluble P-Selectin

Washed platelets were pretreated with DMSO 0.3%, ASA (0.3 mmol/L), or oil extract (0.1 and 1 mg/mL) for 15 min at 37°C and then stimulated by thrombin (2 U/mL) for 45 min at 37°C. Supernatants were collected after centrifugation (2.000 g, 10 min, 4°C) and stored at −70°C until sP-selectin analysis (Invitrogen Corporation, CA, USA).

### 2.6. Measurement of Platelet Secretion

Secretion was analyzed after preincubation of platelets (480 *μ*L of PRP adjusted to 200 × 10^9^ platelets/L) with 20 *µ*L of DMSO 0.3%, ASA (0.3 mmol/L), or oil extract (0.1 to 1 mg/mL) for 3 min prior to the addition of luciferin/luciferase (50 *μ*L) and agonist (20 *μ*L) (ADP 8 *µ*mol/L, collagen 1.5 *μ*g/mL, or TRAP-6 30 *µ*mol/L). Platelet secretion was measured at real time over a 6 min period in a lumi-aggregometer (Chrono-Log, Havertown, PA, USA) at 37°C with stirring (150 g). Controls were run with DMSO 0.3% and the average control secretion was taken as 100%.

### 2.7. Platelet Aggregation Assay

Platelet aggregation was monitored by light transmission according to Born and Cross [16], using a lumi-aggregometer. Briefly, 480 *μ*L of PRP (200 × 10^9^ platelets/L) was preincubated with 20 *μ*L of DMSO 0.3%, ASA (0.3 mmol/L), or oil extract (0.1 to 1 mg/mL) for 3 min. Thereafter, 20 *μ*L of agonist (ADP 8 *µ*mol/L, collagen 1.5 *μ*g/mL, or TRAP-6 30 *µ*mol/L) was added and platelet aggregation was registered during 6 min. All measurements were performed in triplicate. The results of platelet aggregation (maximal amplitude (%)) were determined by the software AGGRO/LINK (Chrono-Log, Havertown, PA, USA). Controls were run with DMSO 0.3% and the average control aggregation was taken as 100%.

### 2.8. Effect of SQ22536 on ADP-Induced Platelet Aggregation

To elucidate whether the oil extract antiaggregant activity was mediated by the adenylyl cyclase pathway, PRP was pretreated with SQ22536 (200 and 400 *µ*mol/L) for 3 min before the addition of oil extract (1 mg/mL). Then, platelet aggregation was challenged by ADP 8 *µ*mol/L. Platelets firstly exposed to SQ22536 and then ADP were used for control purposes.

### 2.9. Analysis of Platelet Rolling and Firm Adhesion under Controlled Flow Conditions

Experiments under flow were performed in a BioFlux-200 flow system (Fluxion, San Francisco, CA, USA) with high shear plates (48 wells, 0–20 dyne/cm^2^) [17]. The microfluidic chambers were coated with 20 *μ*L of collagen 200 *µ*g/mL at a wall shear rate of 200 s^−1^ for 1 hour. The plaque coating was allowed to dry at room temperature (RT) for one hour. Thereafter, channels were perfused with phosphate buffered saline (PBS) for 10 min to remove the interface and blocked with BSA 5% for 10 min (RT, wall shear rate 200 s^−1^). Whole blood, anticoagulated with sodium citrate, was labeled with calcein-AM (4 *μ*mol/L) and incubated with DMSO 0.3%, PGE_1_ (0.02 mmol/L), or oil extract (1 mg/mL) for 15 min (RT) before being added into the inlet of the well. Chambers were perfused for 10 min at room temperature at a wall shear rate of 1000 s^−1^. The plates were mounted on the stage of an inverted fluorescence microscope (TE200, NIKON, Japan).

Platelet deposition was observed and recorded at real time with a CCD camera (QICAM, QIMaging, Surrey, BC, Canada). Real-time visualization between platelets and collagen interactions was performed using a bright field and fluorescence microscopy. Platelets and collagen interaction over a 30 s interval was defined as rolling whereas platelet immobilization for more than 30 s was defined as firm adhesion. For each flow experiment, fluorescent images were analyzed off-stage by quantifying the area covered by platelets with the ImageJ software (version 1.26t, NIH, USA).

### 2.10. Leukocyte Rolling over Collagen-Bound Platelet Monolayer under Controlled Flow

The collagen-coated microfluidic chambers were rinsed in PBS buffer and perfused with whole blood (anticoagulated with sodium citrate), labeled with calcein-AM 4 *μ*mol/L at a shear rate of 200 s^−1^ for 10 min at RT. Under these conditions, a homogeneous carpet of spread platelets was formed. The remaining blood was washed out with DMSO 0.3%, PGE_1_ (0.02 mmol/L), or oil extract (1 mg/mL) for 3 min. Thereafter, leukocytes rolling and firm adhesion were visualized on the platelet layer with an inverted fluorescence microscope. To this end, leukocytes were previously labeled with rhodamine 6G (50 *μ*L of 0.05%). Digital movies were captured, and translocation velocity was calculated by image analysis (ImageJ) [18].

### 2.11. *In Vivo* Murine Model of Thrombosis

Mice (12–16 weeks old) were anesthetized with a combination of tribromoethanol (270 mg/kg) and xylazine (13 mg/kg). The mesentery was exposed by performing a central incision in the abdomen, permitting the visualization of thrombus development in mesenteric vessels. Thrombosis was induced by injection of 50 mg/kg rose bengal through the tail vein followed by illumination of the exposed mesenteric area with a 1.5 mW green light laser (532 nm). Blood flow was monitored for 60 min and stable occlusion was defined as a blood flow of 0 mL/min for 3 min. DMSO 0.3% (control group, *n* = 3), ASA (200 mg/Kg, *n* = 3), or oil extract (200 mg/kg, *n* = 3), was administered intraperitoneally 30 min before the experiment. Rectal temperatures were similar and within the physiological range among all experimental animals throughout the experimental period.

### 2.12. Statistical Analysis

Data were analyzed using SPSS version 17.0 (SPSS, Inc., Chicago, IL, USA. and expressed as mean ± standard error of mean (SEM). Three or more independent experiments were performed for the different assays. Fifty-percent inhibitory concentration (IC_50_) of oil extract against agonist-induced platelet function was calculated by a regression analysis. Differences between groups were analyzed by Student's paired or unpaired *t*-test and one-way analysis of variance (ANOVA) using Tukey's post hoc test. *P* values <0.05 were considered significant.

## 3. Results

### 3.1. Fatty Acid Determination by Gas Chromatography

The values obtained by gas chromatography for the chemical composition of fatty acids in the oil extract are presented in Table 1. It was observed that the oil extracted from peels of *Mauritia flexuosa* fruits had a high content of saturated fatty acids (59% of C_8_–C_22_), unsaturated fatty acids (37.9% of C_16_–C_18_), and a volatile fraction, which corresponds to limonene (2.7%). Within the composition of saturated fatty acids highlights the presence of isopalmitic (32%) and Stearic (19.8%) acids, while unsaturated fatty acids include oleic (33.4%) and linoleic (3.7%) acids.

In addition to the identification and relative quantification of fatty acid methyl esters the presence of monoterpene limonene was detected. Its compound has a time retention and relative abundance of 4.755 min and 2.7%, respectively. Despite the drastic conditions for the formation of methyl esters such as high temperatures and basic conditions, limonene was not degraded due to its high boiling point (175°C), also is responsible for flavour of the oil extracted from peels of *Mauritia flexuosa *fruits.

### 3.2. Effect of Oil Extract on sP-Selectin Platelet Release

**Figure 1:**
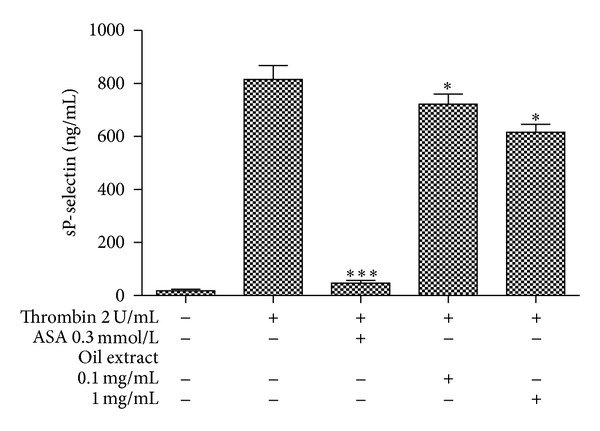
Effect of oil extract on platelet sP-selectin release. Release of platelet sP-selectin was measured after stimulation with thrombin. The graph depicts the mean ± SEM of *n* = 3 experiments. ASA (0.3 mmol/L) was used as positive control. *P < 0.05 and ***P < 0.001.

Pretreatment of washed platelet with increasing concentrations of oil extract (0.1 and 1 mg/mL) significantly inhibited thrombin-induced sP-selectin expression up to 0.1 mg/mL. Concretely, thrombin-induced sP-selectin release was inhibited by 18 ± 2 and 29 ± 3% (*P* < 0.05), in the presence of oil extract at 0.1 and 1 mg/mL, respectively (Figure 1).

### 3.3. Effects of Oil Extract on Platelet ATP Secretion

**Figure 2:**
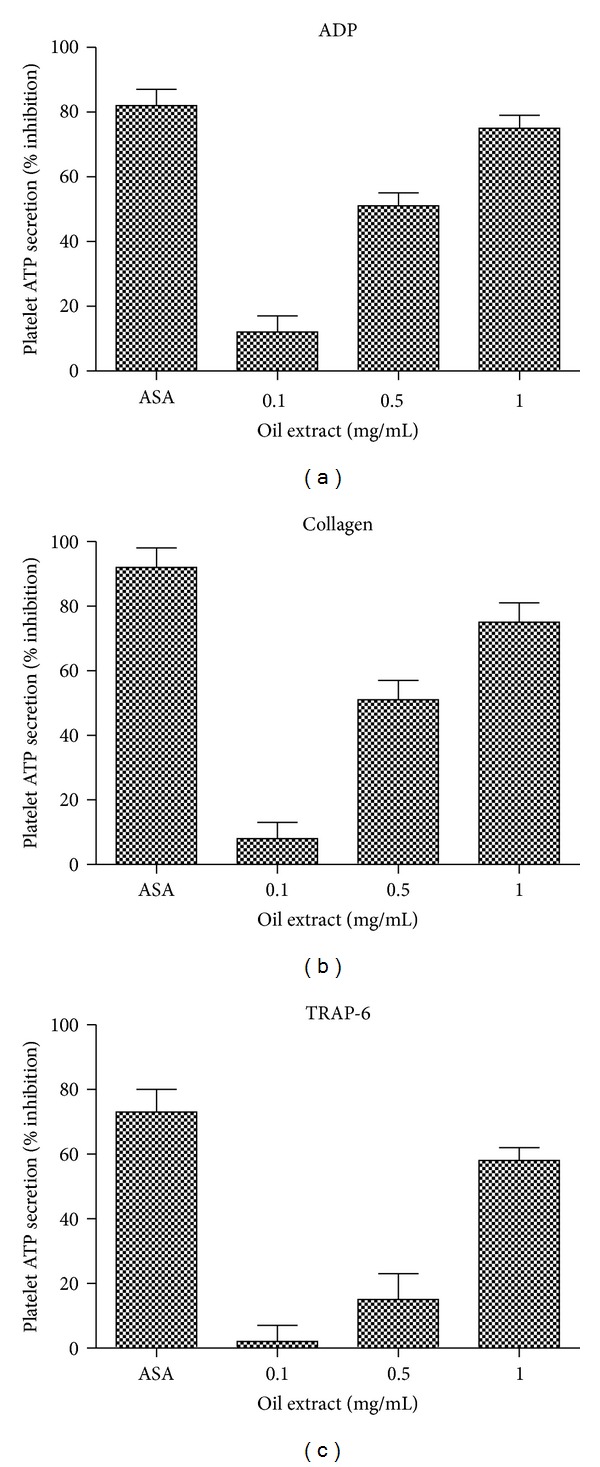
Effect of oil extract on ADP (8 *µ*mol/L), collagen (1.5 *μ*g/mL), and TRAP-6 (30 *µ*mol/L) induced platelet ATP secretion. ASA (0.3 mmol/L) was used as positive control. Results were expressed as % inhibition (mean ± SEM, *n* = 3).

The effects of oil extract on platelet ATP secretion induced by ADP, collagen, and TRAP-6 are shown in Figure 2. Oil extract showed inhibitory effects on ADP-, collagen-, and TRAP-6-induced platelet ATP secretion (*P* < 0.05). Oil extract inhibited ADP-induced ATP secretion, with a calculated IC_50_ concentration of 0.59 mg/mL. Similarly, the IC_50_ concentration for oil extract on collagen-induced platelet ATP-secretion was about 0.69 mg/mL, while the IC_50_ concentration for oil extract on TRAP-6-induced platelet ATP-secretion was about 0.93 mg/mL.

### 3.4. Effects of Oil Extract on Platelet Aggregation

**Figure 3:**
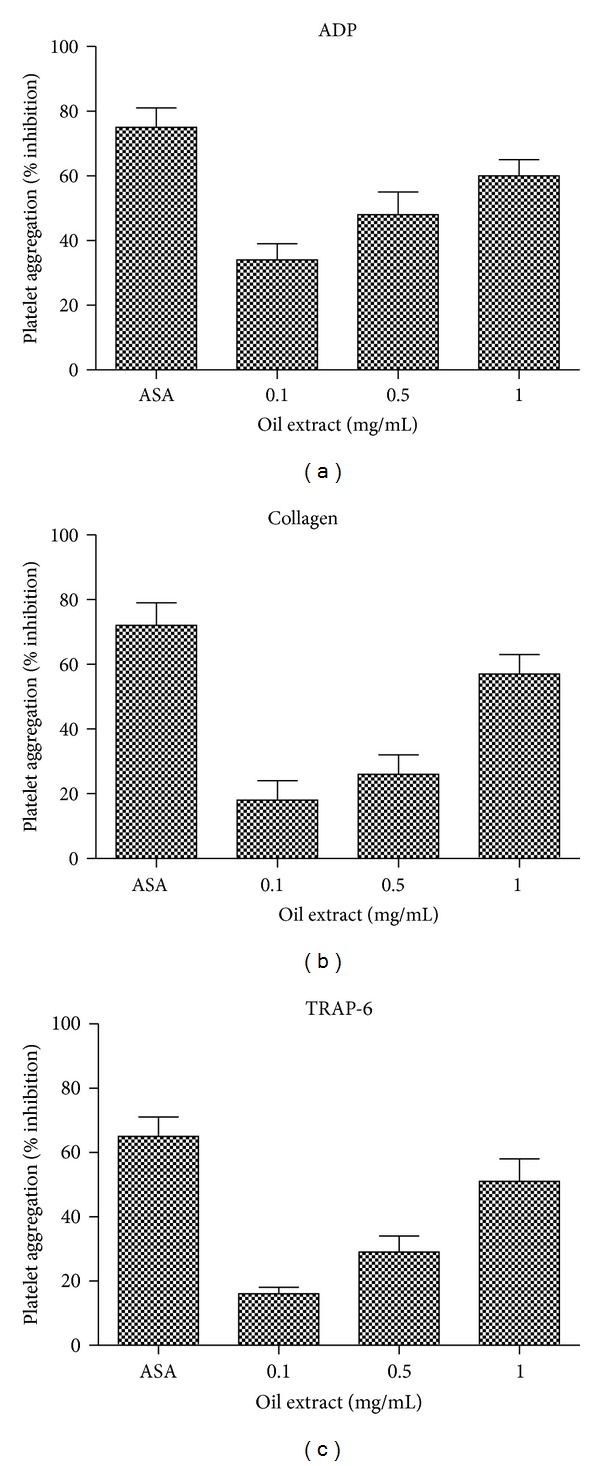
Quantitation of the inhibitory effect of oil extract on platelet aggregation induced by ADP (8 *µ*mol/L), collagen (1.5 *μ*g/mL), and TRAP-6 (30 *µ*mol/L). ASA (0.3 mmol/L) was used as positive control. Results were expressed as % inhibition (mean ± SEM, *n* = 3).

The effects of oil extract on ADP-, collagen-, and TRAP-6-induced platelet aggregation are presented in Figure 3. Oil extract showed inhibitory effects on ADP-, collagen-, and TRAP-6-induced platelet aggregation (*P* < 0.05). Oil extract effectively reduced ADP-induced platelet aggregation with a 50% inhibitory concentration (IC_50_) of 0.65 mg/mL. Similarly, oil extract also suppressed collagen- and TRAP-6-induced platelet aggregation with IC_50_ of 0.93 and 0.99 mg/mL.

### 3.5. SQ22536 Did Not Attenuate Effect of Oil Extract on Platelet Aggregation

To study if the inhibition of platelet function by oil extract is thought to be mediated by the stimulation of adenylate cyclase with increased intraplatelet cAMP concentrations, we incubated platelet with SQ22536 200 and 400 *µ*mol/L (an adenylate cyclase inhibitor) and then with oil extract for platelet aggregation induced by ADP. We found that SQ22536 could not reverse the inhibitory effect of oil extract on platelet aggregation induced by ADP (*P* > NS). As control, SQ22536 400 *µ*mol/L alone did not exert any effect on ADP (8 *µ*mol/L)-induced platelet aggregation.

### 3.6. Oil Extract Reduces Platelet Spreading on Immobilized Collagen under Flow Conditions

**Figure 4:**
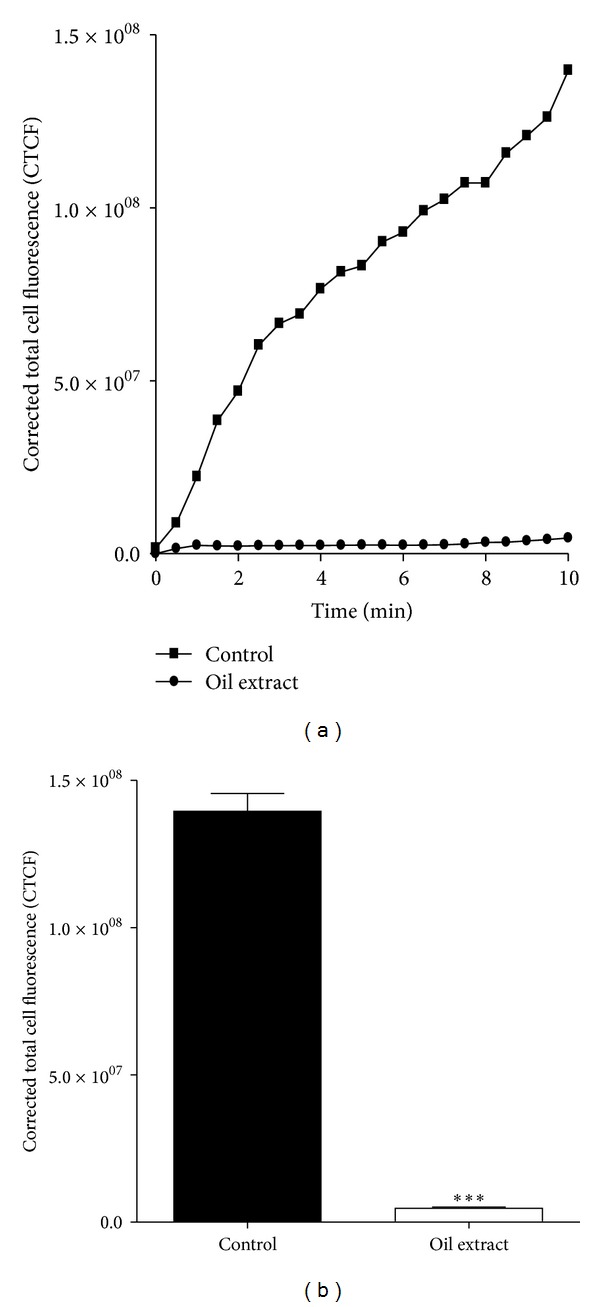
Effects of oil extract on collagen-induced platelet adhesion and aggregation under arterial flow conditions. Citrate-anticoagulated blood was preincubated with DMSO 0.3% (control) or oil extract (1 mg/mL) for 15 min and then was perfused over plaque-coated surfaces for 10 min at room temperature at a shear rate of 1000 s^−1^. (a) shows the intensity (CTCF) over a time lapse. (b) Bar diagram (values are mean ± SEM; *n* = 3). ***P < 0.001. We eliminated the background (yellow colored oil) with plugin that implemented ImageJ's (subtract background command) to avoid quenching phenomena for the yellow colored oil (the Amazonian oil).

The effects of oil extract on platelet adhesion/aggregation to immobilized collagen under arterial flow conditions are shown in Figure 4. After perfusion of citrate-anticoagulated blood over collagen coated plaque surfaces at 37°C with a wall shear rate of 1000 s^−1^ for 10 min, rapid platelet adhesion and aggregate formation were observed (Figure 4). Oil extract (1 mg/mL) reduced collagen-induced platelet adhesion and aggregate formation under controlled flow by 91 ± 2% (*P* < 0.001) (Figure 4). Meanwhile, PGE_1_ (0.02 mmol/L) presented an inhibition by 81 ± 4% (*P* < 0.001).

### 3.7. Oil Extract Reduces Platelet Rolling and Firm Adhesion

**Figure 5:**
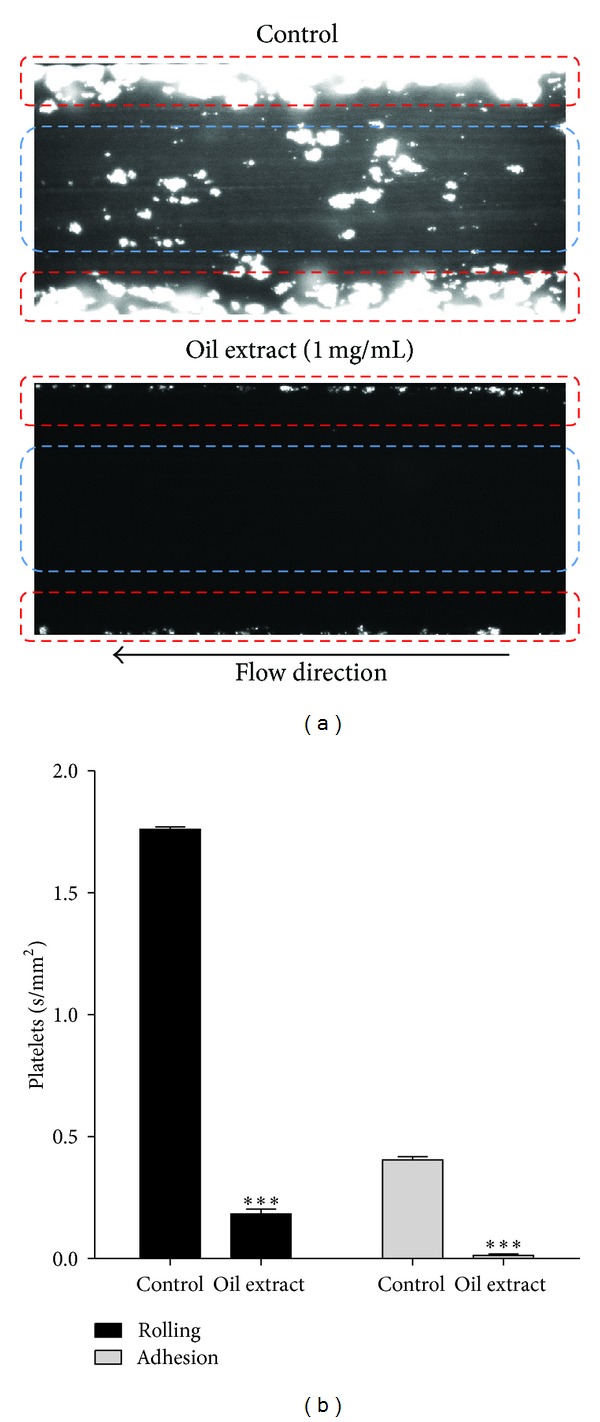
Effect of oil extract on platelet rolling and firm adhesion on collagen under flow conditions. (a) Representative video image of oil extract on platelets rolling and firm adhesion: platelet rolling identified by blue circles and firm adhesion by red circles. (b) Oil extract (1 mg/mL) inhibited platelets rolling and firm adhesion on collagen. Results were expressed as mean ± SEM of *n* = 3. ***P < 0.001. We eliminated the background (yellow colored oil) with plugin that implemented ImageJ's (subtract background command) to avoid quenching phenomena for the yellow colored oil (the Amazonian oil).

Oil extract attenuated interactions between platelet and collagen at both rolling and firm adhesion levels (Figure 5(a)). Thus, platelet rolling in the presence of oil extract (1 mg/mL) was inhibited from 1.76 ± 0.2 in the control group to 0.2 ± 0.1 mm^2^/s (*P* < 0.001). Similarly, in the presence of oil extract (1 mg/mL), platelet firm adhesion on the collagen surface was significantly lower than that on control (Figure 5(b)).

### 3.8. Effect of Oil Extract on Platelet-Leukocyte Interactions

**Figure 6:**
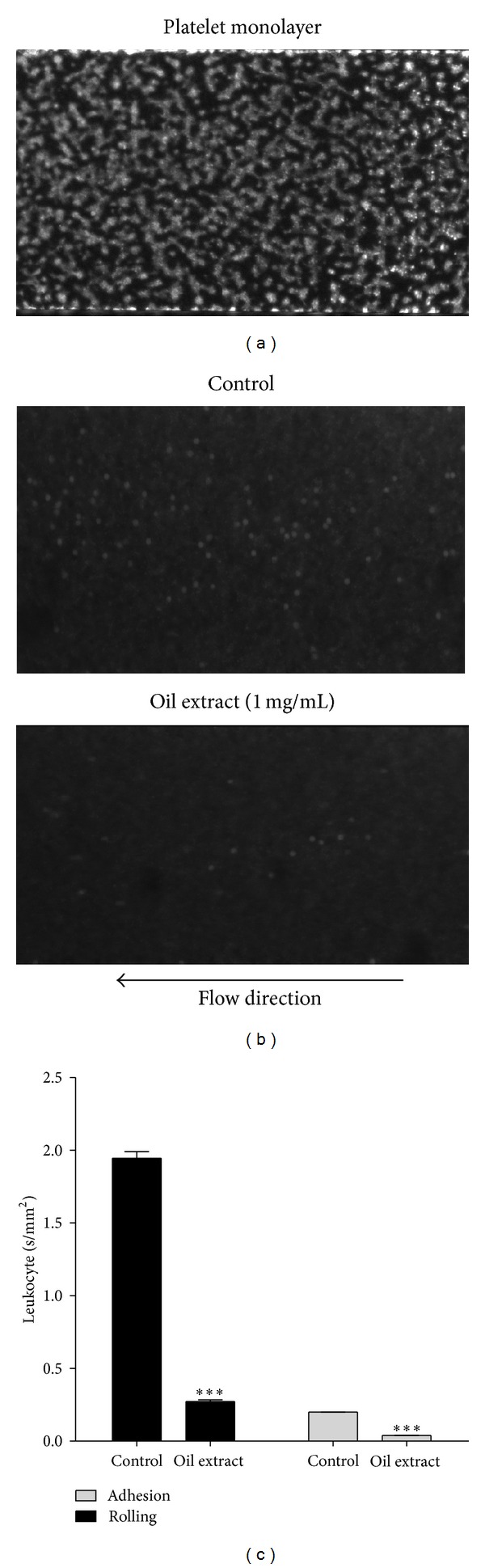
Oil extract reduces leukocyte rolling and firm adhesion over collagen-bound platelet monolayers. (a) Homogeneous carpet of spread platelets labeled with calcein-AM formed on the collage. (b) Representative images of rhodamine 6G labeled leukocyte rolling and adhesion on platelet monolayer at shear rate of 150 s^−1^ in absence (DMSO 0.3%) or presence of oil extract (1 mg/mL). (c) Velocities of leukocyte rolling and firm adhesion on platelet monolayer surface. Results were expressed as mean ± SEM of *n* = 3. ***P < 0.001. We eliminated the background (yellow colored oil) with plugin that implemented ImageJ's (subtract background command) to avoid quenching phenomena for the yellow colored oil (the Amazonian oil).

Results of the real-time analysis of oil extract effects on platelet-leukocyte interactions are shown in Figure 6. Under a shear stress of 150 s^−1^, leukocytes rolled and got attached to activated platelet but not to collagen surfaces (data not shown). Oil extract attenuated interactions between leukocytes and platelet surface, reflecting a reduction of rolling and less adhesion (Figure 6(b)). As shown in Figure 6(c), rolling velocity of leukocytes under a shear stress of 150 s^−1^ over immobilized activated platelets was diminished in the presence of oil extract (1 mg/mL) from 1.9 ± 0.2 in the control group to 0.26 ± 0.1 mm^2^/s (*P* < 0.001). Similarly, oil extract resulted in lower leukocyte firm adhesion on the surface of the platelet monolayer as compared to the control group (*P* < 0.001) (Figure 6(c)).

### 3.9. Effect of Oil Extract on Arterial Thrombus Formation *In Vivo*

**Figure 7:**
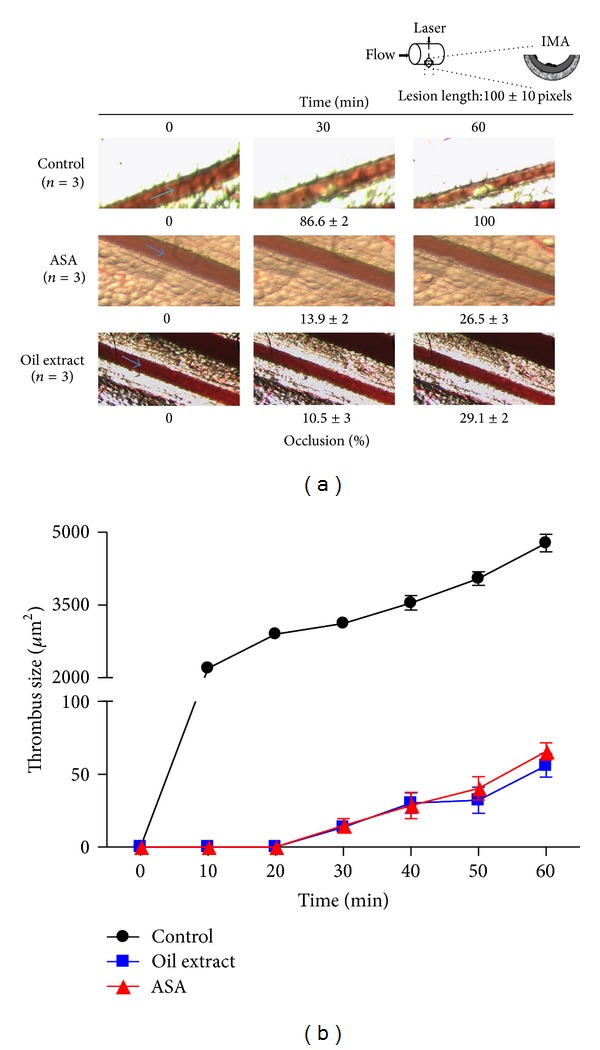
Oil extract inhibits laser-induced thrombus formation in mesenteric artery of C57BL/6 mice. (a) Representative images of thrombus formation at baseline, 10, 20, 30, 40, 50, and 60 min after laser-injured vascular injury in mouse mesenteric artery. (b) Time course changes of thrombus growth rate among the control (DMSO 0.3%), ASA (acetylsalicylic acid), and oil extract for 60 min after laser irradiation. The graph depicts the means ± SEM of *n* = 3 experiments. We eliminated the background (yellow colored oil) with plugin that implemented ImageJ's (subtract background command) to avoid quenching phenomena for the yellow colored oil (the Amazonian oil).

To examine the *in vivo* antithrombotic activity of oil extract, we evaluated the effects of oil extract on laser-injured thrombus formation in mice mesenteric artery *in vivo*. As shown in Figure 7, in untreated mice (control), mesenteric artery was totally blocked by a stable bulky thrombus at 40 min. In contrast, further analysis revealed that the time to form the artery thrombosis (>2500 *µ*m^2^) was drastically prolonged in oil extract-treated mice compared to the mice receiving the same volume of vehicle (Figure 7). Thus, one intraperitoneally bolus injection of oil extract (200 mg/kg) 30 min before laser injury prevented thrombus formation over 60 min after laser injury by 71 ± 2% (*P* < 0.001). Currently ASA is the gold standard for secondary prevention of stroke of vascular origin; therefore, we compared the antithrombotic role of oil extract with ASA. Under our conditions both oil extract (200 mg/kg) and ASA (200 mg/kg) exhibiting the same antithrombotic efficacy (Figure 7), especially when vessel occlusion is evaluated.

## 4. Discussion

In the present study we have demonstrated for the first time that oil extract of fruit from *Mauritia flexuosa* possesses antiplatelet activity and inhibits *in vivo* thrombus formation. In this way, many studies have provided evidence of the protective role of healthy diets in the prevention of CVD [19, 20]. More specifically, a number of dietary components including some fats, polyphenols, and nucleosides have shown to diminish platelet activation [21, 22]. To this respect, our group has recently isolated and identified adenosine and guanosine as a bioactive compound in *Solanum lycopersicum* (a cherry type tomato) with potent antiplatelet activity [21, 23].

Fruit from the palm *Mauritia flexuosa* for its high nutritional value (fat 38%, fiber 30%, carbohydrate 28%, and protein 5%) is a fundamental part of the human diet. Its fruit also contains vitamin A, potassium, calcium, iron, magnesium, and the highest carotenoid contents [24, 25]. In Venezuela indigenous people use this fruit as bread [26], and in Brazil and Ecuador during times of flooding it is the biggest source of food. Currently, fruits of the palm *Mauritia flexuosa* are one of the Amazon fruits more commercialized (pulp, soda, lollipops, ice cream, jams, and yoghurt) [27, 28]. Limonene is a main component of citrus essential oils which are commonly extracted from their matrix by using distillation and it plays an important role in the field of flavours and fragrances for many years thanks to its physical and chemical properties [29, 30].

In the past 2 decades, views about dietary unsaturated fatty acids have moved from speculation about their functions to solid evidence that they not only are essential nutrients but also may favorably modulate many diseases [31]. Thus changes in dietary fatty acids may have mild, but significant, effects on eicosanoid production, platelet aggregation, and blood pressure [32, 33]. Fatty acid determination by gas chromatography in the walnut of fruits from the palm *Mauritia flexuosa *evidenced the presence of unsaturated fatty acids: oleic (27%) and linoleic (22.6%) (data not shown) [34]. Therefore, according to this study, the peel and walnut have a high content of oleic acid.

With respect to antiplatelet and antithrombotic activities, we firstly analyzed the potential antiplatelet properties of the oil extract. The oil extract inhibited platelet secretion and aggregation elicited by a broad range of agonists (Figures 2 and 3). Furthermore, we observed that oil extract prevents platelet rolling and firm adhesion onto collagen under flow conditions.

Thrombin-induced sP-selectin release from human platelets does not seem to be the target pathway of the oil of interest. In addition, platelets are activated by different activators via complex signal pathways. Therefore, we speculate that oil extract may block a common signal pathway involved in platelet activation without participation of adenylyl cyclase pathway (studied by SQ22536). These healthy activities of oil extract from peels of *Mauritia flexuosa *fruits that prevent CVD are mainly due to the presence of unsaturated fatty acids (oleic, linoleic, and palmitoleic acids). In this sense, it has been reported that PPARs agonists (unsaturated fatty acids), which involve an elevation of cAMP, involve subsequent inhibition of platelet function against various agonists [35–37]. In this way, oleic acid is capable of affecting platelet function [38]. Oleic acid is a potent inhibitor of phospholipase A2 which would explain its generally low activity in human platelet extracts and its marked increase of activity during the course of enzyme purification [39]. In addition, linoleic acid present in both the peel and walnut from *Mauritia flexuosa* was shown to inhibit the formation of arterial thrombosis, the expression of tissue factor, and platelet aggregation [37]. These properties are directly related to the prevention of thrombi development occurring in stroke.

Taking into consideration all of these findings, we further provided *in vivo *evidence of such *in vitro*-related oil extract antithrombotic effects. Indeed, using a murine model of real-time thrombus formation, we demonstrate that oil extract inhibited arterial thrombus growth at the same concentration of aspirin, a widely used antiplatelet agent. We even demonstrate, that oil extract administration diminished thrombus growth with kinetics of thrombus inhibition similar to that of aspirin.

The broad range of antiplatelet and antithrombotic effects found in the oil extracted from *Mauritia flexuosa* may render this functionally active principle in potent inhibitors of platelet function with a potential preventive effect on thrombus formation.

## 5. Conclusion

The data presented herein demonstrate, for the first time to our knowledge, the protective effect of oil extract from *Mauritia flexuosa* on platelet activation and thrombosis formation. These effects, in terms of primary prevention, could modify cardiovascular risk without any of the side effects normally associated with antiplatelet drugs. Moreover, *Mauritia flexuosa* may constitute a functional ingredient adding antiplatelet activities to processed foods.

## Figures and Tables

**Table 1:** Fatty acid compositions of oil extract from peel of *Mauritia flexuosa. *

Fatty acid composition (%)	Peel
8:0 (caprylic acid)	1.0
10:0 (capric acid)	0.3
12:0 (lauric acid)	0.7
13:0 (tridecylic acid)	0.2
14:0 (myristic acid)	1.5
15:0 (valeric acid)	0.5
16:0 (isopalmitic acid)	32.0
16:0 (palmitic acid)	1.0
14:0 (margaric acid)	0.8
18:0 (stearic acid)	19.8
20:0 (arachidic acid)	0.8
22:0 (behenic acid)	0.4
16:1 (palmitoleic acid)	0.8
18:1 (oleic acid)	33.4
18:2 (linoleic acid)	3.7
